# Effect of High-Fat Diet upon Inflammatory Markers and Aortic Stiffening in Mice

**DOI:** 10.1155/2014/914102

**Published:** 2014-06-11

**Authors:** Andre Bento Chaves Santana, Thais Cristina de Souza Oliveira, Barbara Lobo Bianconi, Valerio Garrone Barauna, Ed Wilson Cavalcante Oliveira Santos, Tatiana P. Alves, Juliane Cristina S. Silva, Patricia Fiorino, Primavera Borelli, Maria Claudia Costa Irigoyen, José Eduardo Krieger, Silvia Lacchini

**Affiliations:** ^1^Institute of Biomedical Sciences, University of Sao Paulo, 05508-000 Sao Paulo, SP, Brazil; ^2^Heart Institute, University of Sao Paulo Medical School, 05403-900 Sao Paulo, SP, Brazil; ^3^Faculty of Pharmaceutical Sciences, University of Sao Paulo, 05508-000 Sao Paulo, SP, Brazil; ^4^Health, Biology and Science Center, Mackenzie University, 01302-907 Sao Paulo, SP, Brazil

## Abstract

Changes in lifestyle such as increase in high-fat food consumption are an important cause for vascular diseases. The present study aimed to investigate the involvement of ACE and TGF-**β** in the aorta stiffness induced by high-fat diet. C57BL/6 male mice were divided in two groups according to their diet for 8 weeks: standard diet (ST) and high-fat diet (HF). At the end of the protocol, body weight gain, adipose tissue content, serum lipids and glucose levels, and aorta morphometric and biochemical measurements were performed. Analysis of collagen fibers by picrosirius staining of aorta slices showed that HF diet promoted increase of thin (55%) and thick (100%) collagen fibers deposition and concomitant disorganization of these fibers orientations in the aorta vascular wall (50%). To unravel the mechanism involved, myeloperoxidase (MPO) and angiotensin I converting enzyme (ACE) were evaluated by protein expression and enzyme activity. HF diet increased MPO (90%) and ACE (28%) activities, as well as protein expression of ACE. TGF-**β** was also increased in aorta tissue of HF diet mice after 8 weeks. Altogether, we have observed that the HF diet-induced aortic stiffening may be associated with increased oxidative stress damage and activation of the RAS in vascular tissue.

## 1. Introduction

Aortic disease is an important cause of mortality worldwide, which may be exemplified by vascular aneurysm, atherosclerotic lesion, and vascular stiffening [1]. Lifestyle characteristics such as obesity promote adverse effects on the vascular system by increasing aortic stiffness [2].

Increased collagen deposition leads to stiffening of the arterial wall, compromising vascular distensibility and contributing to a feedback of the hypertensive process [3]. The tunica adventitia (the outmost layer of the vessels) is composed of connective tissue whose main matrix component is collagen fibers. Some lamellae of elastic fibers can also be found in this network [4]. The outer layers of the tunica adventitia are rich in collagen types I and III which are produced by local fibroblasts [5]. In light microscopy, these layers are not distinct but integrate with the connective tissue around the outside of the vessel, helping it to maintain its vascular structure. The deposition of collagen has a functional role in the adventitia maintaining, strengthening the vessel wall to prevent its rupture. Collagen is abundantly deposited in extracellular matrix (ECM) acting in maintaining the integrity and resistance of vascular wall [6].

Arterial stiffening involves mechanisms of ECM remodeling, promoted by increased collagen deposition, and this mechanism involves both endothelial cells from intima and fibroblasts from adventitia layer. Macrophages, neutrophils, T cells, and adipocytes can also induce arterial stiffness by paracrine stimuli [7]. Oxidative stress can also promote aortic stiffness, resulting in remodeling of the vascular wall [8]. Some studies have showed that during the processes of aorta stiffening in hypertensive patients the renin-angiotensin system may be overactivated [9].

The renin-angiotensin system (RAS) is described as a cascade of biochemical reactions whose activity is essential for cardiovascular homeostasis. Angiotensin II (Ang II) is a main effector of the RAS acting on homeostasis and cardiovascular function, through the type 1 (AT1) and type 2 (AT2) receptors [10]. Angiotensin I (Ang I) is converted to Ang II by the action of angiotensin I-converting enzyme (ACE) expressed mainly in the pulmonary vascular endothelium. The local RAS may act on the vasculature independently of systemic RAS [11].

Ang II at the vascular wall is capable to induce the tunica intima hyperplasia regardless of hemodynamic or neurohumoral factors [11–13]. The development of vascular pathologies such as atherogenesis, hypertension, and restenosis is the result of trophic stimulation of the vessel wall, primarily promoted by the action of Ang II, leading to structural modifications in vascular ECM [11, 12, 14].

In addition, Ang II stimulates the activation of several genes that lead to changes in vascular function, including matrix metalloproteinases (MMPs), monocyte chemoattractant protein 1 (MCP-1), vascular cell adhesion molecule (V-CAM), vascular endothelial growth factor (VEGF) and plasminogen activator inhibitor-1 (PAI-1), interleukins, tumor necrosis factor alpha (TNF-*α*), and transforming growth factor beta (TGF-*β*) [15]. The TGF-*β* is a multifunctional protein, participating in the regulation of cell division, differentiation, migration, cell adhesion, extracellular matrix production, and others, involved in several pathologies, including cardiovascular diseases [16].

Perivascular tissue can exert paracrine actions though the release of proinflammatory agents as interleukin-6 (IL-6) and TNF-*α* produced by adipose tissue. Perivascular adipose tissue (PVAT) also expresses components of RAS such as ACE and produces Ang II, contributing to autocrine and paracrine actions in inflammatory responses [17, 18].

In this context, the increased consumption of lipids can promote major changes in arterial tissue, especially through activation of the RAS, the induction of oxidative stress, and proinflammatory factors. In this way, it can induce structural changes in conductance vessels and constitute a risk for developing cardiovascular diseases. Thus, it is of great importance to understand the effects that the high-fat diet (HF) has on the aorta. Furthermore, it is essential to identify markers related to the processes of stiffening of conductance vessels caused by high-fat diet. Moreover, there are known the effects of lipid overload on RAS components and its relationship with local mechanisms of inflammatory sites by the action of TGF-*β*. Thus, the present study aimed to investigate the causal effect of high-fat diet upon vascular inflammatory markers and its relationship to aortic stiffness.

## 2. Methods

### 2.1. Animals

Twenty-six C57BL/6J male mice (8 weeks old) from the Animal House of the Department of Anatomy, Institute of Biomedical Sciences/University of São Paulo (ICB/USP), were used. The animals were kept in a room with light/dark cycle of 12 hours and controlled temperature between 22 and 24°C, with feed and water given* ad libitum*. This study was approved by* Institutional Ethics Committee on the Use of Animals* (CEUA-ICB/USP).

Mice were randomized into 2 groups following their diet for 8 weeks: (1) standard diet (ST, *n* = 10) [19] and (2) high-fat diet [20] (HF, *n* = 16). Animals were weighed weekly, and at the end of the eight weeks, the animals were subjected to six-hour fasting before blood withdrawals for serum glucose and lipid profile analyses. After that, adipose tissue was quantified; heart rate and blood pressure were measured previously to blood and tissue collections.

### 2.2. Quantification of Adipose Tissue

X-ray images were captured for quantification of adipose tissue in each group by* In-Vivo Imaging System FX PRO* in Center for Research Facilities (CEFAP)—ICB/USP. The images obtained were used in measurements of adipose tissue from each animal using the software ImageJ (version 1.32j, from NIH) and* ICY* (version 1.3.6.0, from http://www.bioimageanalysis.org/). The percentage of visceral adipose tissue content was obtained by measuring periepididymal and retroperitoneal cushions relative to the body image of each animal.

### 2.3. Heart Rate and Systolic Blood Pressure Measures

Heart rate (HR) and systolic blood pressure (SBP) were obtained using a tail-cuff plethysmography (Kent Scientific) coupled to an analogic-to-digital converter. HR and SBO signals were analysed by using* BP-*2000* Blood Pressure Analysis* program. All mice were adapted to the system for three days before measurements. Measurements were conducted for two days, using at least 10 correct measures in each day, for each mouse.

### 2.4. Blood and Tissue Collections

At the end of the protocol, mice were anesthetized with a lethal intraperitoneal dose of ketamine (180 mg/Kg) and xylazine (20 mg/kg). Blood sample and aorta tissue were harvested and stored in ultrafreezer for later analysis. Tissue collection procedures for biochemical measurements were different from those for histological study. For immunoblotting and enzyme activity assays, mice were perfused with 0.9% NaCl solution and thoracic aorta was harvested and immediately frozen in liquid nitrogen. For histological and immunostaining assays, mice received heparin (50 U per animal, together with anesthesia) and were perfused with 0.9% NaCl solution at constant pressure (80–90 mmHg) followed by buffered 4% formalin solution. The aortae were postfixed in 4% buffered formalin for 24–48 hours. Tissues were processed and paraffin-embedded for histological evaluation.

### 2.5. Serum Glucose, Cholesterol, HDL, LDL, and Triglycerides

Serum glucose, cholesterol, HDL, LDL, and triglyceride levels were measured by spectrophotometer according to the procedures described in commercially available kits (Labtest).

### 2.6. Angiotensin Converting Enzyme Activity Assay

ACE activity in aortae extracts was determined using Abz-FRK(Dnp)P-OH derivatives as substrates by continuously measuring the fluorescence according to our previous publications [21, 22]. Tissue samples were quickly harvested, homogenized in Tris-HCl buffer, pH 7.0, containing 50 mM NaCl, and centrifuged at 1,000 g for 10 min. The assays were performed at 37°C in 0.1 M Tris-HCl buffer, pH 7.0, containing 50 mM NaCl and 10 mM ZnCl_2_. Hydrolysis rate of the intramoleculary quenched fluorogenic substrate Abz-YRK-(Dnp)p (10 mM) incubated with aliquots of tissues homogenate and serum for 30 min at 37°C was assessed to obtain ACE enzymatic activity. Fluorescence increments along the time were read at 420 nm emission: 320 nm excitation. Tissue ACE activity was expressed as fluorescence units (AFU)·min^−1^·mg^−1^ of protein [21, 22]. The protein content was determined by the Bradford method by using bovine serum albumin as the standard (Bio-Rad protein assay).

### 2.7. Myeloperoxidase Enzyme Activity Assay

The myeloperoxidase (MPO) enzyme activity was measured by continuous recording using the reagent* Amplex Ultrared*. The enzyme activity was measured continuously every 1 minute in a fluorometer by fluorescence emission at 530 nm wavelengths emission and 590 nm excitation. Approximately 50 *μ*L of supernatant for each sample was incubated with 50 *μ*L of substrate* Amplex*. The enzymatic activity measurement of MPO was performed by using as inhibitor sodium azide (10 *μ*M). The MPO activity was expressed as fluorescence units (AFU)·min^−1^·mg^−1^ of protein. All assays were performed in duplicate.

### 2.8. Immunoblotting Analysis

Aortae were lysed in RIPA buffer (1 mM EDTA, 1 mM EGTA, 2 mM MgCl_2_, 5 mM KCl, 25 mM Hepes, pH 7.5, 2 mM DTT, 1 mM PMSF, 0.1% Triton X-100, and 1 : 100 cocktail of protease inhibitors) and stirred for 30 minutes at 4°C. The homogenates were centrifuged at 10000 ×g for 10 min at 4°C. After that, the supernatant was collected. Tissue lysate (50 *μ*g) was heated in sample buffer (200 mM Tris-HCl, pH 6.8, 40% glycerol, 8% sodium dodecyl sulphate, 0.1% DDT, and 0.4% bromophenol blue) at 100°C for 5 minutes. Next, the samples were subjected to sodium dodecyl sulfate polyacrylamide gel electrophoresis (SDS-PAGE) using the* Mini-PROTEAN Tetra Cell* (Bio-Rad). After electrophoresis, the gel proteins were transferred to nitrocellulose membrane* Hybond-C Extra* (GE Healthcare) in transfer medium (0.025 M Tris, 0.192 M glycine, 0.1% SDS, and 20% methanol) using the system* Trans-Blot SD Semi-Dry Transfer Cell* (*Bio-Rad*). The membranes were washed in a solution of 1X TBS-T three times (5 minutes each wash). Next, the membranes were blocked with 5% bovine serum albumin (BSA) for 3 hours, then washed three times in TBS-T, and then incubated overnight with antibody anti-ACE (anti-ACE goat polyclonal C-20, SC-12187, Santa Cruz Biotechnology, Inc.) in 1 : 500 dilution, antibody anti-TGF-*β* (anti-TGF-beta 1 rabbit polyclonal SC-146, Santa Cruz Biotechnology, Inc.) in 1 : 500 dilution, and anti *β*-tubulin (anti-*β*-tubulin, Santa Cruz sc-9104) in 1 : 1000 dilution. Membranes were exposed in* ECL WB Detection Reagents* (GE Healthcare) and revealed in* Image Quant LAS 4000 mini* (GE Healthcare) equipment. Protein bands were quantified by optical densitometry using* ImageJ* software (version 1.32j, NIH).

### 2.9. Histological Preparation of Aortae Sections

The aortae maintained in buffered 4% formalin for 24–48 h to complete the fixation process were processed and paraplast (Fisher Brand) embedded for histological evaluation. Descending thoracic aorta cross slices were obtained using a microtome (*Microm HM200 ERGOSTAR*) with 5 *μ*m thickness. Three to five slices for each animal were placed on each slide.

### 2.10. Immunostaining Assays

Immunohistochemistry (IHC) was performed using the ABC (streptavidin-biotin-peroxidase) method and used the following antibodies: ACE (SC-12187, Santa Cruz Biotechnology, Inc., 1 : 500 dilution) and TGF-*β* (SC-146, Santa Cruz Biotechnology, Inc., 1 : 500 dilution). Antigen retrieval was performed using citrate buffer pH 6.0 for TGF-*β* unmasking. The blocking of endogenous peroxidase was done with 3% H_2_O_2_ solution and incubation of the slices for 30 minutes at room temperature. Histological sections of the aorta were incubated at 4°C with primary antibodies diluted in blocking solution (3% bovine serum albumin in PBS) for a minimum of 18 hours (overnight). Afterwards, incubation was performed with secondary antibody conjugated with biotin and streptavidin conjugated with horseradish peroxidase (HRP). Then, 3,3-diaminobenzidine (DAB) was used as chromogen. Counterstaining was performed with hematoxylin. Negative control reactions were prepared omitting the primary antibody in one slice for each aorta.

### 2.11. Morphometric Analysis

Morphometric measurements and collagen fibers analysis were performed on picrosirius-stained tissues. The cross-sections from aortae were viewed in microscope (*Axio Imager.A2, Zeiss*), and the images were digitized by computer image analysis system (AxioVision release 4.8.2 SP2).

In morphometric measurements, intima, media, and adventitia areas were calculated. All measurements were performed using* ImageJ* program. In these analyses, three slices were examined in each animal, obtained in three distinct sections of the thoracic aorta. The intima area was obtained from the difference between the area inside the inner elastic lamina and the luminal area. The media area was obtained from the difference between the area inside the outer elastic lamina and the area inside the inner elastic lamina. Lastly, the adventitia area was obtained from the difference between the area around of the adventitia and the area inside the outer elastic lamina. Values obtained were expressed as the ratio between adventitia/media area and the ratio between media/intima area.

### 2.12. Quantification of Collagen Fibers

Evaluation of collagen fibers was performed in cross-sections from the aorta by use of polarized light. This analysis aims to assess the deposition of thin and thick fibers of collagen in the extracellular matrix and assess how these fibers are arranged in the vessel due to the high fat diet. The study of molecular distribution of collagen fibers was made in picrosirius-stained sections. Therefore picrosirius-polarization method is specific for collagenous structures and enhances their birefringence quality [23]. This characteristic permits, under polarized light, the observation of thin collagen fibers with weak birefringence in green color. Already thick collagen fibers are composed of bundles of highly birefringent fibers presented with orange or red color [24].

The quantification of aortic collagen fibers was performed in three distinct sections of the aorta, by using* ICY* program and the plugin* KMeans Color Quantization*. Values obtained were expressed in the graph from the area of thick and thin collagen fibers in adventitia. In these analyses, three 5 *μ*m slices were examined per animal for each aortic slice.

### 2.13. Measurement of the Waviness of Collagen Fibers

The analysis of the waviness of the collagen fibers in polarized light was performed by program* ImageJ* using the* Orientation J plugin* [25, 26]. The quantification of waviness was made to verify the degree of disorganization of collagen fibers, measuring the entropy of each region of interest (ROI) marked in the micrograph. Again, three slices were examined per animal, always considering the same relative position of image in the vessel. For each segment of collagen at 400x magnification five entropy measures were performed. In each section of the aorta four distinct segments in cross-section were analyzed. Values obtained were expressed in graph from the percentage of entropy.

### 2.14. Statistical Analysis

Experimental values are evaluated as mean ± standard deviation. The results are statistically evaluated by comparing the groups by unpaired *t*-test with* Welch* correction. The test was used to determine if there were differences in the phenomena observed between the groups ST versus HF. For all analyses the significance level of *P* ≤ 0.05 was assumed.

## 3. Results

### 3.1. Effects of High-Fat Diet on Body Weight (BW), Adipose Tissue Content, Heart Rate (HR), Systolic Blood Pressure (SBP), and Lipid and Glucose Levels

HF diet induced greater body weight gain compared with mice in ST diet after 8 weeks (Table 1). HF diet group increased the BW by 12% compared to the beginning of the protocol while no significant weight gain was observed in the ST group.

**Figure 1:**
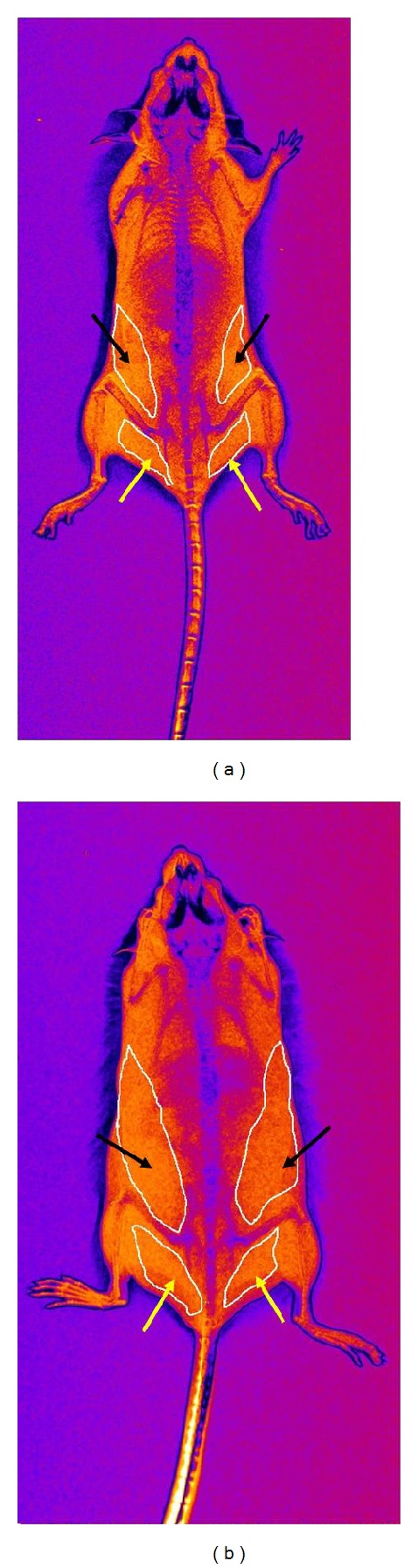
X-ray imaging with filter for viewing adipose tissue (marked in white). Black arrows show the retroperitoneal cushions of adipose tissue. Yellow arrows show the periepididymal cushions of adipose tissue. (a) ST diet mice; (b) HF diet mice.

Adipose tissue content was analyzed by X-ray imaging system. The increase in periepididymal and retroperitoneal adipose tissues in the HF diet group can be noticed from the representative image (Figure 1). Quantification of body fat mass at the end of 8 weeks of diet showed significant difference between groups, with total adipose tissue mass increased in HF diet group (Table 1). These data demonstrate the efficiency of HF diet to promote fat gain in wild-type mice. Also, serum lipid profile showed significantly increased levels of total cholesterol, LDL, and glucose in HF diet group when compared to the ST diet group (Table 1). Differences were not observed in serum HDL and VLDL between groups. Heart rate was not different among groups while systolic blood pressure decreased in HF diet group compared to the ST diet group (Table 1).

### 3.2. HF Diet Induces Structural Adaptation in Aorta

**Figure 2:**
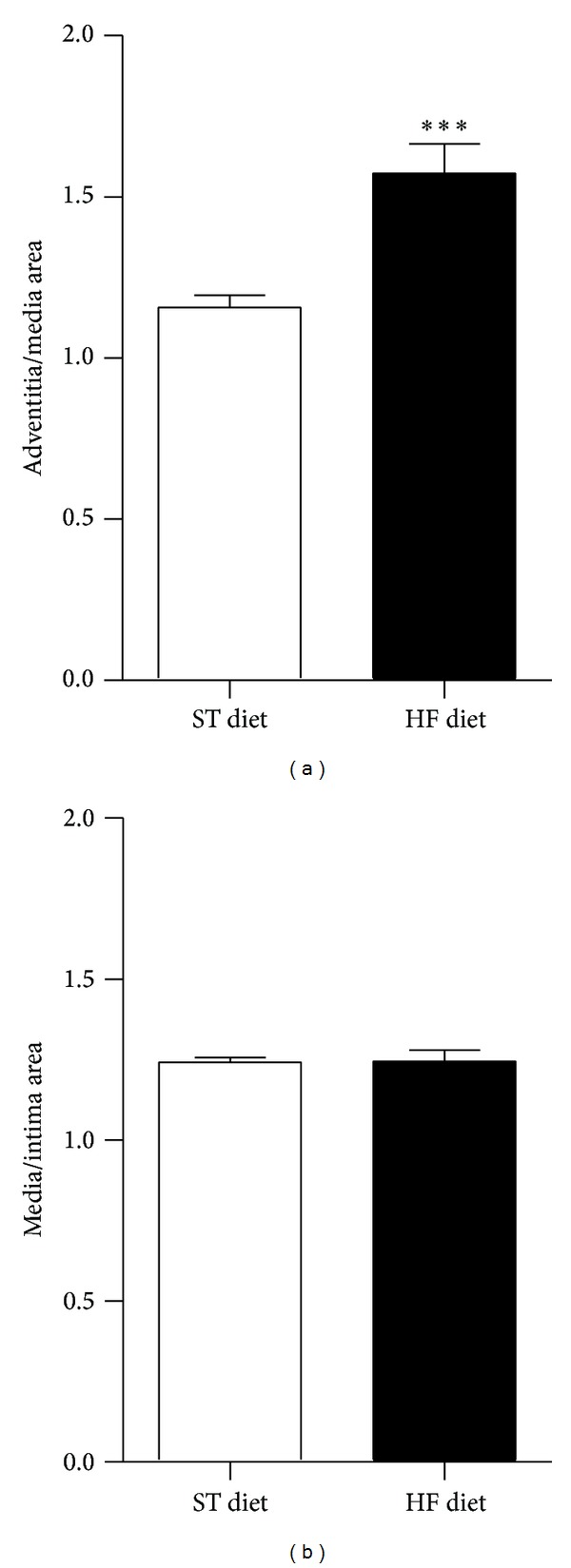
Morphometric analyses of aorta sections. Adventitia area/media area (a); media/intima area (b). ***P ≤ 0.001; *n* = 5 per group.

**Figure 3:**
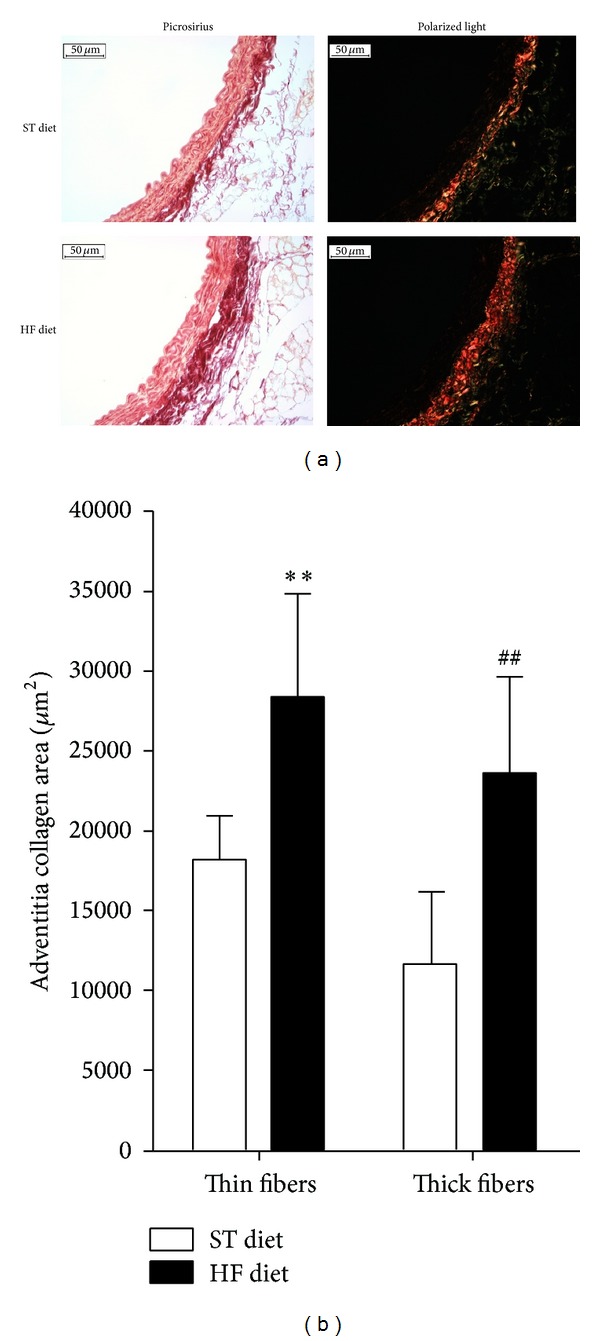
Collagen content in aortae. Representative photomicrographs of mouse aorta under picrosirius staining-light microscopy and polarized light microscopy, magnification of 400x (a). Quantitative analyses of the collagen fibers in adventitia from the HF diet and ST diet groups (b). **P ≤ 0.01; ^##^ *P* ≤ 0.01; HF diet versus ST diet; *n* = 5 per group.

Morphometric analyses of thoracic aorta segments showed significant difference in the tunica adventitia/tunica media area ratio after the 8-week protocol (ST diet, 1.16 ± 0.04 versus HF diet, 1.57 ± 0.09; *P* < 0.001; Figure 2(a)). However, difference was not found in the tunica media/tunica intima area ratio (ST diet, 1.24 ± 0.02 versus HF diet, 1.25 ± 0.04, Figure 2(b)). Picrosirius staining of aorta slices showed an increase in collagen deposition in the tunica adventitia of HF diet group (Figure 3(a)). Under polarized light it was observed that the total collagen content increase was due to a twofold increase in thick fibers (ST, 11630 ± 4489 *μ*m^2^ versus HF, 23519 ± 6033 *μ*m^2^; *P* < 0.01; red staining) and a 1.5-fold increase in thin fibers (ST, 18155 ± 2737 *μ*m^2^ versus HF, 28209 ± 6587 *μ*m^2^; *P* < 0.01; green staining) in HF diet group compared to ST diet group (Figures 3(a) and 3(b)).

**Figure 4:**
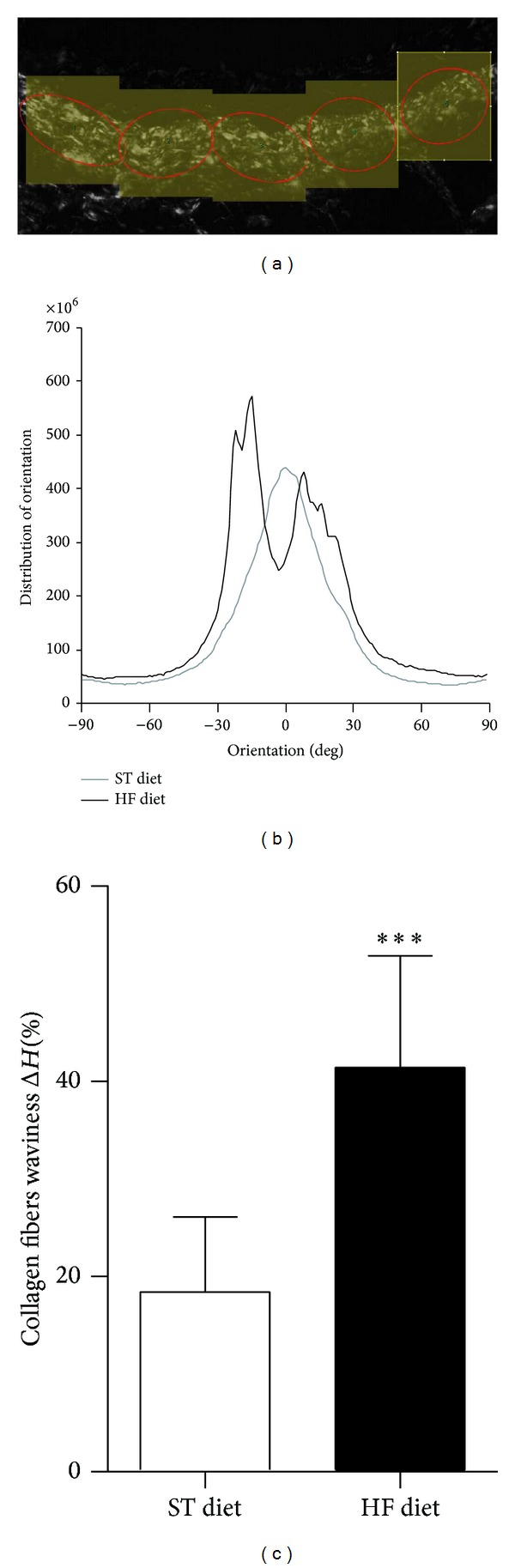
Quantitative analyses of collagen fibers orientation and waviness. Representative image of waviness quantifications of collagen fibers in the tunica adventitia performed using the* ImageJ.* Photomicrograph at 400 times magnification under polarized light (a). The overall fibers orientation represented as a normalized histogram (b). Waviness analysis showed increased disorientation of collagen fibers in the tunica adventitia of the aortas of mice maintained on HF diet (c). Δ*H* is the entropy of the fibers; ***P ≤ 0.001; *n* = 5 per group.

Waviness quantification of collagen fibers (Figure 4(a)) in the tunica adventitia showed a significant increase in entropy of fibers disposition in HF diet group. The average entropy of fibers of the ST diet group was 18.4 ± 7.7%, while the HF diet group obtained 41.4 ± 11.4% (Figures 4(b) and 4(c)).

### 3.3. Aortic ACE and MPO Activity after High-Fat Diet

**Figure 5:**
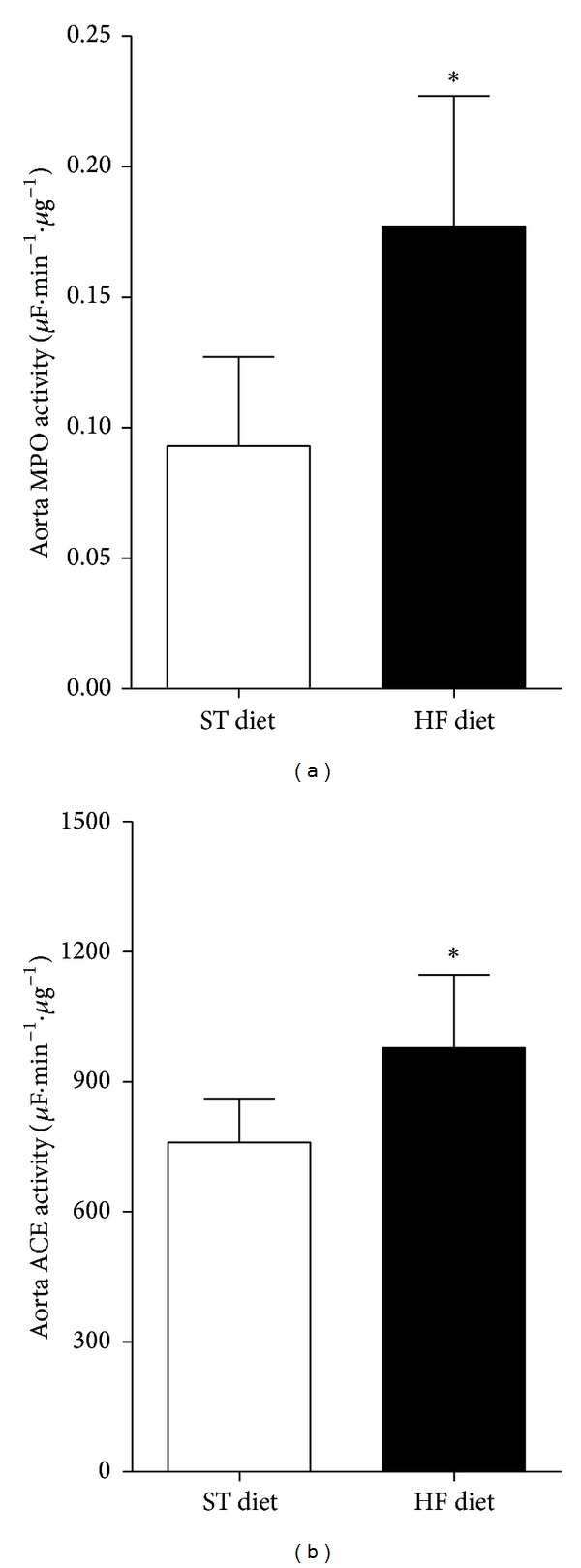
Myeloperoxidase (MPO, (a)) and angiotensin I converting enzyme (ACE, (b)) activities in aortic tissue. *P ≤ 0.05; *n* = 6 per group.

HF diet increased MPO activity by 90% (ST, 0.0929 ± 0.0342 versus HF, 0.1770 ± 0.0500 *μ*F·min^−1^·*μ*g^−1^, *P* < 0.01, Figure 5(a)) and ACE activity by 28% (ST, 759.9 ± 101.2 versus HF, 978.2 ± 168.3 *μ*F·min^−1^·*μ*g^−1^, Figure 5(b)).

**Figure 6:**
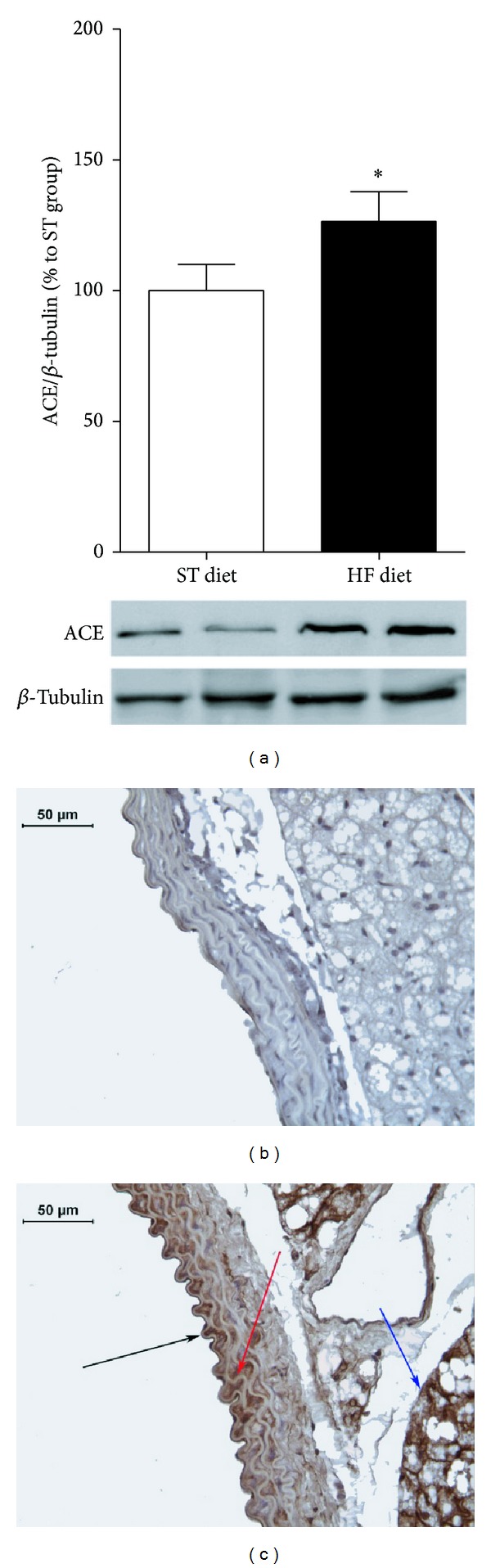
ACE expression analyses by immunoblotting (a) and immunostaining in aortae (ST diet (b) and HF diet (c)). ACE expression was normalized by *β*-tubulin expression. In (c), black arrow indicates ACE expression in tunica intima; red arrow indicates ACE expression in tunica media; blue arrow indicates ACE expression in perivascular adipose tissue. *P ≤ 0.05; *n* = 8 per group.

ACE protein expression analyzed by immunoblotting was also increased by 26.5% after 8 weeks of HF diet (Figure 6(a)). Immunostaining of aortae slices showed higher ACE signal in intima, media, and perivascular adipose tissue in the HF diet group (Figure 6(c)).

### 3.4. Increased TGF-*β* Expression in HF Diet Group

**Figure 7:**
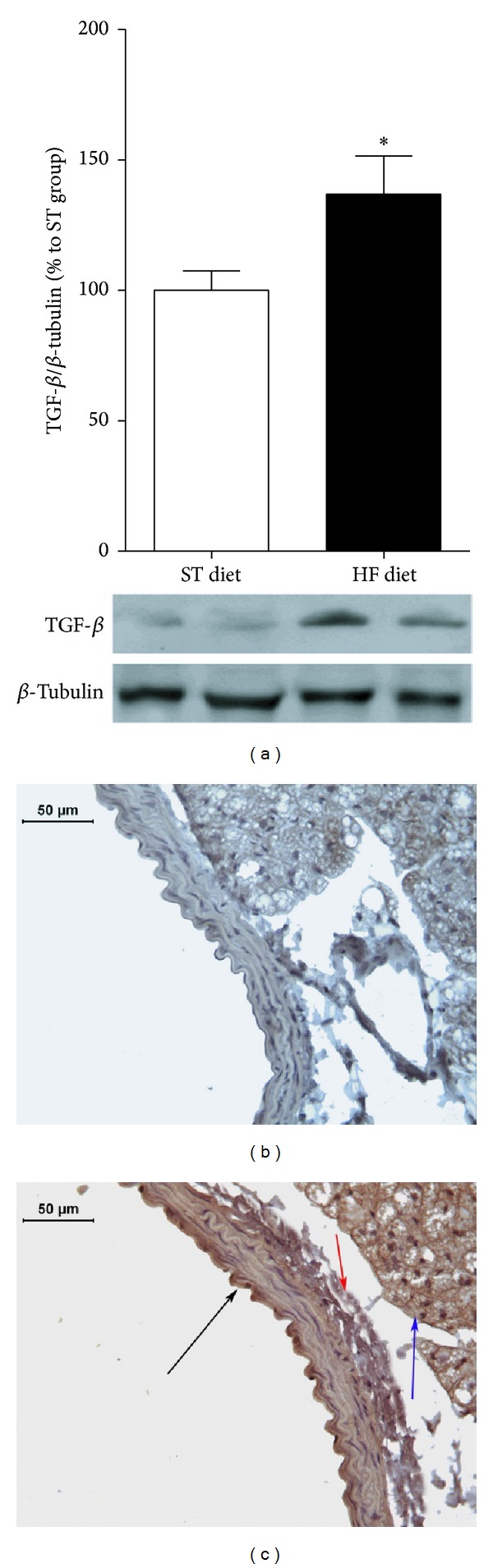
TGF-*β* expression analyses by immunoblotting (a) and immunostaining in aortae (ST diet (b) and HF diet (c)). TGF-*β* expression was normalized by *β*-tubulin expression. TGF-*β* expression was normalized by *β*-tubulin expression. In (c), black arrow indicates TGF-*β* expression in tunica intima; red arrow indicates TGF-*β* expression in tunica adventitia; blue arrow indicates ACE expression in perivascular adipose tissue. *P ≤ 0.05, *n* = 8 per group.

Aortic TGF-*β* protein expression in HF diet group was also increased compared to the ST diet group (Figures 7(a)–7(c)). By immunoblotting, the HF diet group showed an increase of 36.5% compared to the ST diet group. By immunostaining aorta slices, TGF-*β* showed intense staining in the intima, media, and also perivascular adipose tissue (Figure 7(c)).

## 4. Discussion

The results of the present study reveal that both local RAS and proinflammatory markers such as MPO and TGF-*β* may be related to the vascular structural changes induced by the high-fat diet for 8 weeks in wild-type mice.

Images obtained by X-ray scanning showed a greater accumulation of adipose tissue in animals fed the high-fat diet compared to the control group. Accumulation of retroperitoneal and epididymal fat in mice is equivalent to visceral adipose tissue of human obesity [27, 28]. The type of consumed lipid can modify the accumulation of adipose tissue. There is a high correlation between the percentage of body fat and percentage of saturated fat and monounsaturated fat intake by diet.

The increased consumption of high-fat diet, associated with decreased physical activity and endocrine disorders and genetic and metabolic effect may have a role in the development of obesity. These processes can influence the metabolism of fatty acids, as well as inducing an increase of adipose tissue accumulation and production of proinflammatory cytokines, among other humoral factors [29]. The elevation of serum LDL cholesterol is accompanied by excess of visceral adipose tissue and contributes to increasing the risk of developing dyslipidemia [30].

In this study, we have verified an increase in the tunica adventitia/tunica media area ratio, suggesting a possible increase in perivascular collagen deposition. Under polarized light microscopy an increase in both thick and thin collagen fibers in mice subjected to high-fat diet was confirmed. In addition, the evaluation of collagen fibers waviness showed an increase of entropy, suggesting not only an increase of collagen fibers deposition but also a loss of parallel fibrillar distribution, leading to a possible increase in the distribution network of collagen fibers and a consequent increase in the resistance of vascular wall, as recently reported [31]. This increase of vascular wall resistance may be the starting trigger to the arterial stiffening process.

Several mechanisms may be related to arterial stiffening in obesity, including hypertension, oxidative stress, growth factors, and inflammation. Interestingly, the group that received high-fat diet did not develop hypertension. In contrast, these mice showed a reduction of blood pressure. This reduction in blood pressure may be due to the metabolic modifications, where the group which received high-fat diet developed hyperlipidemia and hyperglycemia. In this context, the reduction of blood pressure in rats and mice with diabetes mellitus was already described [32].

Regarding the role of oxidative stress, growth factors, and inflammation, we have evaluated the myeloperoxidase activity as a marker of leukocyte activity. It has already been shown that the increase of MPO activity in aortae of high-fat diet mice is an important indicator of local oxidative stress mediated by leukocytes triggered by accumulation of lipids [33]. In fact, our study demonstrated increase of aortic MPO activity in high-fat diet group. It is well described that oxidative stress contributes to important changes in vascular function, especially in endothelial reactivity and injuries caused by dyslipidemia [34]. It is important to note that a certain degree of peroxidation is important to eliminate the accumulation of LDL in vascular wall by monocyte activity [35].

Hyperglycemia, hyperlipidemia, and oxidative stress lead to endothelial activation and an increase of other proteins involved in inflammation and growth cell. In this case, ACE and TGF-*β* are important mechanisms leading to cellular growth, inflammation, and extracellular matrix deposition. It was verified that patients with visceral obesity present high levels of free fatty acids, which promote the development of diabetes and endothelial dysfunction [36, 37]. Also, polymorphonuclear and mononuclear cells are capable of producing Ang II through the action of free fatty acids to promote insulin resistance and endothelial dysfunction [38].

Considering that high-fat diet could promote substantial changes in RAS activity, this study verified an increase of ACE expression and activity in aorta of high-fat diet mice. Increased enzymatic activity of ACE in aorta suggests an increase of angiotensin II production that is able to induce collagen synthesis in vascular smooth muscle cells via AT1 receptor and stimulate the release of TGF-*β* [39, 40]. In addition, Ang II has a role in the remodeling of the ECM increasing collagen synthesis and deposition and reducing its degradation [41].

Positive staining for ACE in aorta by immunostaining was observed mainly in tunica intima and perivascular adipose tissue of high-fat diet mice group. These data indicate that vascular RAS may be capable of starting and maintaining proinflammatory processes, since the production of angiotensin II can act not only on vascular tone but also in inflammatory responses. This inflammatory response could lead to cell proliferation and activation of immune cells such as monocytes.

TGF-*β* plays an important role in vascular remodeling processes, with an important role in profibrotic responses [16]. By immunostaining assays, an intense staining of TGF-*β* in mice aorta submitted to high-fat diet was observed. This technique permits us to verify that TGF-*β* was found not only in the intima and media layers, but also in adventitia and perivascular multilocular adipose tissue.

The increased deposition of collagen fibers in the tunica adventitia of mice aortae subjected to a high-fat diet is evident in histological evaluations. The high waviness degree of collagen fibers in the aortic tunica adventitia promoted by high-fat diet is another point of structural change that may be directly or indirectly mediated by local RAS, oxidative stress, or TGF-*β* activity. Thus, these data show that high-fat diet was able to induce stiffness of vascular aorta wall through the increase in deposition and disorganization of thick collagen fibers. The extracellular matrix remodeling depends on three key factors, such as hemodynamic changes and humoral factors triggering the synthesis, release, and activation of substances that can influence the growth, death, or cell migration (release of TGF-*β*), and structural changes in vascular wall. Thus, our results support the idea that aortic stiffness in response to high-fat diet may be dependent on oxidative stress and proinflammatory mechanisms involving RAS.

## 5. Conclusions

This study showed that the stiffness of aortic wall induced by high fat diet involves an increase of deposition and disorganization of collagen fibers in tunica adventitia. This structural change of collagen fibers disposition is accompanied mainly by an increased activity and protein expression of ACE at tunica intima and perivascular adipose tissue. Moreover, the increases of TGF-*β* in the intima, adventitia, and perivascular adipose tissue, associated with the elevation of myeloperoxidase activity, suggest that an inflammatory process, associated with oxidative stress, is even a profibrotic stimulus for the collagen deposition and vascular stiffening.

## Figures and Tables

**Table 1:** Body weight, percentage of adipose tissue, systolic blood pressure, heart rate, HDL, LDL, VLDL cholesterol, and serum glucose levels of mice maintained on different diets.

	ST diet	HF diet
Body weight (g)		
First week	23.1 ± 1.3 (*n* = 10)	23.5 ± 1.8 (*n* = 16)
Eighth week	30.6 ± 3.2 (*n* = 10)	35.1 ± 4.1* (*n* = 16)
Adipose tissue (%)	16.95 ± 1.7 (*n* = 5)	28.33 ± 5.8* (*n* = 10)
Heart rate (bpm)	609 ± 39 (*n* = 9)	624 ± 50 (*n* = 16)
Systolic blood pressure (mmHg)	117.5 ± 10.9 (*n* = 9)	103.8 ± 5.2* (*n* = 16)
HDL (mg/dL)	42.4 ± 7.3 (*n* = 7)	58.8 ± 9.3 (*n* = 7)
LDL (mg/dL)	86.37 ± 17.9 (*n* = 7)	112.9 ± 24.3* (*n* = 7)
VLDL (mg/dL)	12.7 ± 1.6 (*n* = 7)	11.3 ± 2.3 (*n* = 7)
Cholesterol (mg/dL)	141.5 ± 23.2 (*n* = 7)	182.9 ± 32.0* (*n* = 7)
Glucose (mg/dL)	170.2 ± 17.6 (*n* = 7)	372.0 ± 53.5* (*n* = 7)

*P ≤ 0.05.
